# Robust Estimation of Recent Effective Population Size from Number of Independent Origins in Soft Sweeps

**DOI:** 10.1093/molbev/msz081

**Published:** 2019-04-09

**Authors:** Bhavin S Khatri, Austin Burt

**Affiliations:** 1 Department of Life Sciences, Imperial College London, Ascot, Berkshire, United Kingdom; 2 The Francis Crick Institute, London, United Kingdom

**Keywords:** effective population size, soft sweeps, recurrent mutation, demographic oscillations, *Anopheles*

## Abstract

Estimating recent effective population size is of great importance in characterizing and predicting the evolution of natural populations. Methods based on nucleotide diversity may underestimate current day effective population sizes due to historical bottlenecks, whereas methods that reconstruct demographic history typically only detect long-term variations. However, soft selective sweeps, which leave a fingerprint of mutational history by recurrent mutations on independent haplotype backgrounds, holds promise of an estimate more representative of recent population history. Here, we present a simple and robust method of estimation based only on knowledge of the number of independent recurrent origins and the current frequency of the beneficial allele in a population sample, independent of the strength of selection and age of the mutation. Using a forward-time theoretical framework, we show the mean number of origins is a function of θ=2Nμ and current allele frequency, through a simple equation, and the distribution is approximately Poisson. This estimate is robust to whether mutants preexisted before selection arose and is equally accurate for diploid populations with incomplete dominance. For fast (e.g., seasonal) demographic changes compared with time scale for fixation of the mutant allele, and for moderate peak-to-trough ratios, we show our constant population size estimate can be used to bound the maximum and minimum population size. Applied to the *Vgsc* gene of *Anopheles gambiae*, we estimate an effective population size of roughly $6\times 10^{7}$, and including seasonal demographic oscillations, a minimum effective population size >$3\times 10^{7}$, and a maximum <$6\times 10^{9}$, suggesting a mean $∼10^{9}$.

## Introduction

Studying the differences in sequences between individuals in a population has the potential to give new insight into evolutionary processes: the evolutionary forces of selection, mutation, migration, and drift can leave a signature in the pattern and frequency of polymorphisms in time and space, which population genetic theory can be used to infer (Bollback et al. 2008; Gutenkunst et al. 2009; Liu and Fu 2015; Zanini et al. 2015; Khatri 2016; Petkova et al. 2016; Feder et al. 2017). A key parameter to estimate for any evolving population is the effective population size (Fisher 1930; Wright 1931), as it determines the underlying nature of the evolutionary dynamics and the relative importance of genetic drift versus selection for evolving traits. In particular, having an accurate estimate of recent effective population size has impact on our ability to predict the outcomes of evolution, as the current population size controls the mutational input through the parameter θ=2Nμ and the fate of rare variants in a population via the population scaled strength of selection 2*Ns* (Kimura 1962). However, there is not a single well-defined measure of effective population size and different estimates will depend on the particular evolutionary pressures on the trait or genomic region under consideration, as well as on previous population histories (Charlesworth 2009). A common method to estimate effective population size is from the nucleotide diversity *π* of neutral regions of a genome, where for 2Nμ≪1, we expect π=2Nμ (Charlesworth 2009). This relation represents a balance between mutations introducing variation at rate *μ* and drift removing variation at rate $\frac{1}{2N}$. However, nucleotide diversity will tend to be dominated by population bottlenecks, and so be insensitive to recent population expansions (Karasov et al. 2010), and there is a need for methods to estimate effective population sizes which are more representative of current day census size. Methods based on linkage disequilibrium tend to be limited to small population sizes (Waples and Do 2010). On the other hand, although there are a number of methods that attempt to directly infer demographic history (Pybus et al. 2000; Gutenkunst et al. 2009; Browning and Browning, 2015; Liu and Fu 2015), these methods are either complex and computationally intensive or only able to detect long-term changes in population size. There are currently no methods that simply and robustly allow estimation of very recent effective population sizes.

A recent popular paradigm to study variation in populations is “soft sweeps,” where for sufficiently large population sizes (Nμ≳1), multiple copies of the same mutation, distinguished by their haplotype background, coexist in the population. This provides a direct genetic fingerprint on the rate at which mutations enter a population *θ*, which without their distinguishing haplotype backgrounds would be hidden. Precise information about *θ* is effectively hidden when mutations arise infrequently per generation (Nμ≪1), since in this weak successive mutations regime, a single dominant haplotype fixes in a population before other haplotypes have a chance to establish; these are termed “hard sweeps,” as each subsequent sweep erases any previous information, giving a weak bound that θ≪1. In a series of seminal articles by Pennings and Hermisson (Hermisson and Pennings 2005; Pennings and Hermisson 2006a, 2006b), much of the basic theory of soft sweeps was developed within a coalescence framework. In particular, the mean number and the distribution of independent origins in a neutral population sample were found to be given by Ewens’ sampling framework (Ewens 2010). Recently, using this approach, Anderson et al. (2017) estimated the effective population size of the malaria parasite; such estimates of *N* from soft sweeps should be representative of the effective size over the time period of the sweep (Karasov et al. 2010) and more representative of current day census size. However, estimating the maximum likelihood effective population size requires using Ewens’ formula (Ewens 2010) for the probability of observing a certain number of distinct alleles in a sample of only neutral alleles; although exact it is not very practical for large sample sizes, as it requires evaluating the Stirling number of the first kind, a combinatorial factor that has not been implemented in most programming languages. In addition, when the mutant allele has not yet gone to fixation, we need to account for the fact that samples will contain both wild type and mutant alleles; this requires the extra complication of having to convolve Ewens’ formula with a binomial distribution for the probability of observing a given number of mutants in a sample given the frequency of the mutant. Finally, Pennings et al. (2014) estimated an effective population size of HIV from soft sweeps of $N∼10^{5}$ larger than estimates from nucleotide diversity; however, they used a specific theory for the case of a single amino acid change given by two different nucleotide mutations, so that the two codons give a maximum of two detectable independent origins.

In this article, we present a simple semideterministic forward-time approach, based on a nonhomogeneous Poisson establishment rate of independent mutants, which thereafter grow deterministically (Messer and Neher 2012). We show that this gives very accurate estimates of the number of independent origins as a function of the time since selection sets in. In the haploid case, we show explicitly the likelihood function is independent of the selection coefficient and only dependent on the frequency of the mutant allele and so does not require estimation of the selection coefficient or the age of the allele. This approach has the advantage of being simple to implement, as the likelihood function is a nonhomogeneous Poisson process, and is particularly appealing as the results can be understood in intuitive terms in a forward-time framework. Further, we show the method is robust to whether or not the mutation was preexisting in the population and is equally accurate for diploid populations with incomplete dominance (0<h<1). Finally, we apply our method to recent data from the *Vgsc* locus from the *Anopheles gambiae* 1000 genomes (1000Ag) project (*Anopheles gambiae* 1000 Genomes Consortium 2017) to find an estimate of effective population size almost 2 orders of magnitude greater than is estimated by analyzing nucleotide diversity. Moreover, to account for the marked seasonal population dynamics of this species, we show that it is possible to calculate a bound for the maximum and minimum effective population sizes, based on an estimate of effective population size using the constant population size method.

## Theory

We calculate the likelihood of the number of origins with two assumptions: 1) we assume a nonhomogeneous (time-dependent) Poisson process such that mutant alleles establish with rate α(t)=2Nμs[1−x(t)], where *x*(*t*) is the frequency of all mutant alleles in the population; 2) after establishment of the *k*^th^ mutant allele, its frequency $x_{k}(t)$ increases deterministically. The mean number of origins at time *T* is then determined by calculating the average number of establishment events in a time window 0 to *t_K_*, where *t_K_* is the latest possible time of establishment, such that it can grow deterministically to a critical frequency to be sampled from the population at some time *T*.

### Deterministic Growth

We assume that the overall mutant population grows according to the following differential equation:

(1) $$\frac{dx}{dt}=\mathrm{sx}(1-x)+\mu (1-x),$$

where the first term is the change in frequency due to frequency independent selection (assuming s≪1) and second is the change in frequency due to mutations arising from the wild-type population at mutation rate *μ*. This has the following closed-form solution:

(2) $$x(t)=\frac{\left(sx_{0}+\mu )e^{(s+\mu )t}-\mu (1-x_{0}\right)}{\left(sx_{0}+\mu )e^{(s+\mu )t}+s(1-x_{0}\right)},$$

which in its tanh form is

(3) $$x(t)=\frac{s-\mu }{2s}+\frac{\gamma }{s}\tanh\gamma \left(t-t^{*}\right),$$

where

(4) $$t^{*}=\frac{1}{\gamma }\tanh^{-1}\left(\frac{s-\mu -2sx_{0}}{s+\mu }\right),$$

where γ=(s+μ)/2 and *x*_0_ is the initial frequency of the total mutant population. As in this deterministic framework, the mutant allele only asymptotically reaches fixation as t→∞, we identify $t^{*}$ as the characteristic or typical time to fixation, which is the inflexion point of the tanh function and roughly the point at which the mutant has reached a frequency of (s−μ)/2s≈1/2 for s≫μ; the actual time to fixation with discrete populations and drift will always be of the same order of magnitude as $t^{*}$. Here, we assume that the initial frequency of the mutant is zero and so using the identity $\tanh^{-1}(z)=\frac{1}{2}\ln\left(\frac{1+z}{1-z}\right)$ (|z|<1),

(5) $$t^{*}=\frac{1}{s+\mu }\ln\left(\frac{s}{\mu }\right).$$

We see that the typical time to fixation $t^{*}$ has a logarithmic dependence on the mutation rate and can increase without bound for small mutation rates since we must wait for mutations to arise before selection can act to increase its frequency. Note that our approach here is in contrast to (Karasov et al. 2010; Messer and Neher 2012; Wilson et al. 2014) who typically assume an expression for the mutant frequency which ignores initial conditions and de novo mutation, which as we see can cause a large effect on the time to fixation; in our case, this is important as we require the mutant to have zero initial frequency, when the selection pressure arises.

### Stochastic Establishment and Likelihood of Number of Origins

We assume mutant alleles arise by de novo mutation at a time-varying (nonhomogeneous) rate proportional to the number of wild-type individuals Nμ[1−x(t)]. De novo mutants must reach a critical frequency $x_{\text{est}}∼\frac{1}{2Ns}$ at which point more mutant individuals are added by selection compared with the change in number due to drift (Desai and Fisher 2007). The probability that a de novo mutant, starting at frequency 1/N, grows by drift to size $Nx_{\text{est}}=\frac{1}{2s}$, is just the inverse of the size of this neutral subpopulation, $p_{\text{est}}\approx 2s$. The rate of establishment of mutants is then

(6) α(t)=2Nμs[1−x(t)].

We make the assumption that establishments occur randomly and independently and so the underlying probability distribution for the number of establishments up to time $t_{K}(T)$, the time of establishment of last mutant to possibly be sampled at a latter time *T*, is given by a nonhomogeneous Poisson process:

(7) $$p[\eta (T)|N,s,\mu ]=L[N,s,\mu |\eta (T)]=\frac{\bar{\eta }{\left(T\right)}^{\eta }}{\eta !}e^{-\bar{\eta }(T)},$$

where η(T) is the number of independent origins at time *T*, and where the mean is given by the integral of the rate *α* up to time $t_{K}(T)$:

(8) $$\begin{array}{cc} \bar{\eta }(T) & =\int_{0}^{t_{K}(T)}\alpha (t)dt\\ & =2N\mu \left\{\gamma t_{K}-\ln\left[\frac{\cosh\gamma (t_{K}-t^{*})}{\cosh\gamma t^{*}}\right]\right\}. \end{array}$$

The time of the last establishment $t_{K}(T)$ is straightforward to calculate as shown next.

#### *Calculating* t_K_

The time for the last possible establishment, *t_K_* of the *K*^th^ mutant, in order to be sampled with high probability at time *T*, is calculated by using a deterministic approximation for the change in frequency of the *K*^th^ mutant. In an experiment, and in simulation, individuals of a population are sampled with a sample size *n_s_*; in simulation this is done using multinomial sampling with the allele frequencies determined from simulation. Here, for simplicity, we assume that when a mutant allele frequency is above $x_{s}=1/n_{s}$ then the mutant will be found in a sample of size *n_s_*. With a deterministic time-course of the *K*^th^ mutant, there is a one-to-one correspondence between its frequency at time *T*, $x_{K}(T)$ and the time of establishment *t_K_*, given that its frequency must be $x_{K}(t_{K})=1/2Ns$.

To calculate $x_{K}(t)$, we use the fact that in the deterministic limit the ratio of the frequency of any mutant allele is fixed with respect to the overall mutant population, that is, $x_{K}(t)/x(t)=\text{const}$; this is true whenever the growth function of each mutant is of the same form $\frac{dx_{i}}{dt}=f(x)x_{i}$, which can be proved by showing $\frac{d(x_{i}/x_{j})}{dt}=0$. In this case, once a mutant arises in the population, we assume no more mutations can create the mutant from wild type and that there are no back mutations, so the growth of each mutant follows:

(9) $$\frac{dx_{i}}{dt}=s\left(1-\sum_{j=1}x_{j}\right)x_{i},$$

while the growth of the total number of mutants is given by equation (1); however, once the overall mutant population has established the effect of mutations will be weak compared with selection, as long as s≫μ, and so to a good approximation, the total mutant population also follows the same form as equation (9).

It is then simple to show that the frequency of the *K*th mutant is just a scaling of the frequency of the total mutant population *x*(*t*):

(10) $$x_{K}(t)=\frac{x(t)}{2N\mathrm{sx}(t_{K})},$$

where we have used the fact that at the establishment time *t_K_* we know that the frequency of the mutant must be $x_{K}(t_{K})=1/2Ns$, and that $x_{K}(t)/x(t)=x_{K}(t_{K})/x(t_{K})$. We then solve $x_{K}(T)=x_{s}$, for *t_K_* to give

(11) $$t_{K}(T)=t^{*}+\frac{1}{2\gamma }\text{ln}\left[\frac{2N\mu +x(T)/x_{s}}{2Ns-x(T)/x_{s}}\right],$$

where we have again used the identity ${\text{tanh}}^{-1}(z)=\frac{1}{2}\text{ln}\left(\frac{1+z}{1-z}\right)$ (|z|<1) to arrive at this expression.

#### Simple Expression for Mean Number of Origins

The mean number of origins is calculated by inserting equation (11) into equation (8) and then after some algebra we find:

(12) $$\bar{\eta }(T)=2N\mu \;\text{ln}\left[1+\frac{x(T)n_{s}}{2N\mu }\right],$$

which we see only has dependence on the selection coefficient *s* through the frequency of the total mutant frequency *x*(*T*) at time *T*. This is consistent with the results in Pennings and Hermisson (2006a), where in the coalescence framework they find the probability of a soft sweep in a sample size of 2, at fixation, is independent of the frequency sample path of the mutant allele and weakly bounded by selection through the fixation time. This result suggests that larger sample sizes *n_s_* increase the number of independent origins we should expect to observe. In practice, we can replace *x*(*T*) by the frequency of the mutant in the sample with little error, since it has a weak logarithmic dependence in equation (12), so that $x(T)n_{s}=n_{m}$ is the number of mutants in the sample. Making the standard replacement θ=2Nμ, we arrive at a pleasingly simple expression for the mean number of origins in the sample:

(13) $$\bar{\eta }(T)=\theta \;\text{ln}\left(1+\frac{n_{m}}{\theta }\right),$$

which is only a function of *θ* and the number of mutants *n_m_*.

As shown in the Supplementary Material online, the theory can be extended to the diploid case, where we find an expression for the mean number of origins as a function of the dominance coefficient *h* (assuming incomplete dominance 0<h<1) and the selection coefficient *s*, as well as *N* and *μ*. In this case it is not clear whether the mean number of origins, and hence the Poisson distribution, is independent of the selection parameters *s* and *h*, as the resulting expression is complex. However, as we will see, the haploid expression, with θ=4Nμ in equation (13), is as accurate in the estimation of the effective population size as using the diploid expression, which suggests the dependence on *s* and *h* are weak. In addition, as shown by Pennings and Hermisson (2006a), the probability of a soft sweep has a weak $∼s^{2}$ dependence in diploid populations, which would also suggest a weak dependence on *s* for the number of origins.

## Simulations

### Methods

We simulate the population genetics of multiple recurrent mutations at a single locus using an infinite-alleles Wright–Fisher process. Simulations start assuming a fixed wild type, so that the mutant frequency x(t=0)=0; each subsequent mutation that arises is given its own “allelic” identity to represent it arising on a different haplotype background, and once it enters the population the same allele cannot be produced by mutation from the wild type or any other allele. As is commonly assumed for an infinite-alleles process, we assume in addition there are no back mutations to the wild type. Each mutant allele has the same selective advantage *s* relative to the wild type. For population sizes up to $N=10^{6}$, we use multinomial sampling of alleles with fixed population size *N* to calculate the stochastic change in frequency between generations due to selection and drift. This is replaced by the equivalent multivariate Gaussian distribution with covariance matrix $\langle \Delta x_{i}\Delta x_{j}\rangle -\langle \Delta x_{i}\rangle \langle \Delta x_{j}\rangle =x_{i}(\delta_{\mathrm{ij}}-x_{j})$ for population sizes larger than 10^6^. Correspondence between the two methods was checked for simulations at smaller population sizes (not shown). In both cases, mutations are treated separately and introduced with a nonhomogeneous Poisson process, where the mean number of new mutant alleles in generation *t *+* *1 is given by Nμ[1−x(t)], where *x*(*t*) is the frequency of all mutants in generation *t*; each of these new mutant alleles arise in the population with frequency 1/N (or 1/2N in the diploid case).

At various time points *T*, we sample the vector of frequencies of all independent mutants $x(T)=[x_{1}(T),x_{2}(T),x_{3}(T),\ldots ,x_{K}(T)]$, using multinomial sampling with *K *+* *1 categories (including the wild type, which has frequency $1-\sum_{k=1}^{K}x_{k}$), and sample size *n_s_*. This produces a sample vector n(T), where $n_{k}(T)$ is the number of the *k*^th^ mutant sampled. The number of origins η(T) is then the number of different mutants that are nonzero in the sample.

### Results

**Fig. 1.**
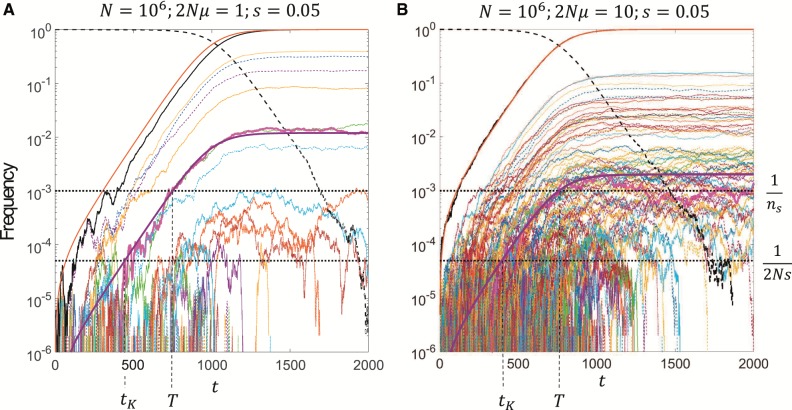
Time series of the frequency of each independent origin of the same recurrent mutant (range of different colors). (*A*) $N=10^{6},\;2N\mu =1$, and *s *=* *0.05, (*B*) same as (*A*), but with 2Nμ=10. Solid black line is the sum of all mutant frequencies ($x(t)=\sum_{k}x_{k}(t)$), dashed black line the frequency of the wild type (1−x(t)), and the solid red line is the deterministic time-course given by equation (3).

In figure 1 is plotted the time series of the frequency of each recurrent mutation from the Wright–Fisher simulations for $N=10^{6}$ and *s *=* *0.05 and two different mutation rates, corresponding to 2Nμ=1 (*A*) and 2Nμ=10 (*B*). We see that at the larger mutation rate there are correspondingly many more mutants in the population, and that the rate of production of mutants is proportional to the frequency of the wild type, signified by the lack of new mutants once the total mutant population has fixed. The red curve is a plot of equation (3), the deterministic solution for the total mutant population over time, and we see that it matches well the time-course found in the simulations, particularly for 2Nμ=10, where stochastic effects of the de novo generation of mutants becomes negligible. The frequency of each of the recurrent mutants follows the same scaling as the total frequency of all mutants, as assumed in the Theory section, and once the mutant population fixes, each of the recurrent mutants plateaus and stops changing in frequency (up to small relative fluctuations), which is as predicted by equation (9). In other words, in the deterministic limit there is a “crowding-out” effect, characteristic of logistic growth, where the growth of a mutant is limited by all other mutants in the population.

In each plot, the highlighted mutant in the thick magenta solid line shows an example of a mutant establishing at the frequency $x_{\text{est}}=1/2Ns$, at time *t_K_*, and then reaching the critical sampling frequency at a time *T*. If *T* is the time of sampling, then this would be the last possible mutant that could contribute to a sample, and the time between 0 and *t_K_* would be the window over which mutants can be generated that could contribute to a sample at time *T*. The distribution of the number of origins at time *T* is just the distribution of the number of establishments in this time window; this is the basis of the semideterministic theoretical calculation of the number of origins described above.

**Fig. 2.**
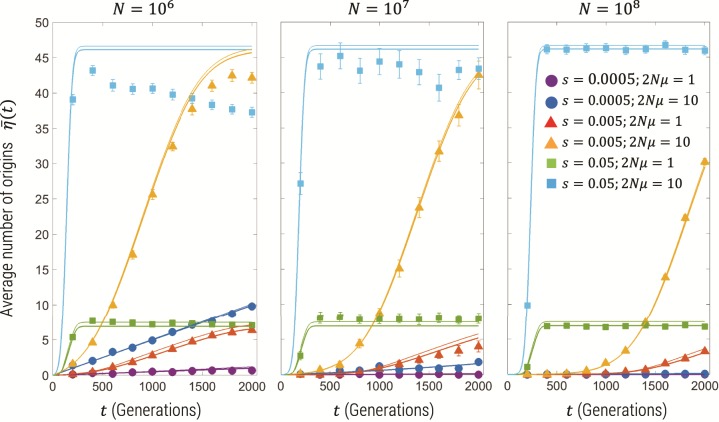
Average number of origins for population sizes of $N=10^{6},\;N=10^{7}$, and $N=10^{8}$. The filled symbols show the simulation results and standard error bars for the parameter combinations shown in the legend; for $N=10^{6}$ and $N=10^{7}$, the simulations used multinomial sampling of the Wright–Fisher drift process with 50 and 10 replicates, respectively, for each parameter combination, whereas for $N=10^{8}$, the multinomial sampling is replaced by the multivariate Gaussian distribution approximation of the drift process (see the Methods section above), where 100 replicates are used in this plot. The solid thick lines are the predictions for the same parameter combination of the semideterministic theory described in this article (Methods), whereas the thin lines represent the prediction of Pennings and Hermisson (2006a), based on Ewens’ sampling theory (Ewens 2010).

**Fig. 3.**
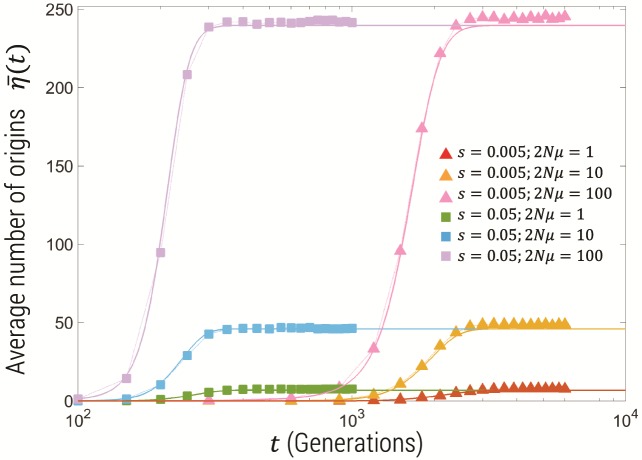
Average number of origins for population size of $N=10^{8}$ on linear-log scale, for 2Nμ={1,10,100} and s={0.05,0.005} showing that plateau number of origins is independent of *s*. The filled symbols show the simulation results and standard error bars for the parameter combinations shown in the legend. The solid thick lines are the predictions for the same parameter combination of the semideterministic theory described in this article (Methods).

Figure 2 shows the results for the mean number of origins $\bar{\eta }(t)$ calculated from simulation (squares), compared the semideterministic theory presented in this article (thick lines) and Pennings and Hermisson’s calculation (thin lines) based on Ewens’ sampling theory (Pennings and Hermisson 2006a; Ewens 2010). We see in general that the time-course of $\bar{\eta }(t)$ reflects the time-course of the frequency of the total mutant population, with a sigmoidal variation, where for the largest selection coefficients we see a plateau reached in <500 generations. Both the semideterministic theory and Ewens’ theory predict that the plateau of $\bar{\eta }(t)$ is independent of the selection coefficient, since $\bar{\eta }(\infty )$ is roughly given by time window over which mutants can be generated, which approximately scales as 1/s, multiplied by the rate of establishment of mutants, which scales like ∼s, cancelling the *s* dependence. This is seen more clearly in figure 3 which is the number of origins plotted for $N=10^{8}$ over a longer timescale for various values of 2Nμ and *s*; we see that the semideterministic theory and the simulations show the plateau is indeed independent of the selection coefficient and only dependent on 2Nμ. We see that the simulations agree with this prediction for the larger population sizes, but for $N=10^{6}$, the number of origins decreases for long times; this is due to drift removing very low frequency variants at the smaller population size, whereas at the larger population sizes drift acts more slowly, such that the change is insignificant on the timescale of the simulation. Finally, we see that the time-course of the mean number of origins before the plateau is different for each population size, where for the smaller selection coefficients the mean number of origins arises more slowly for larger population sizes. This is related to the deterministic time-course of the mutant frequency which, given the initial condition that the mutant frequency is zero, has a strong dependence on the mutation rate as shown by equation (5). The simulations are performed for fixed 2Nμ, and so a larger population size means a smaller mutation rate and so $\bar{\eta }(t)$ increases more slowly.

**Fig. 4.**
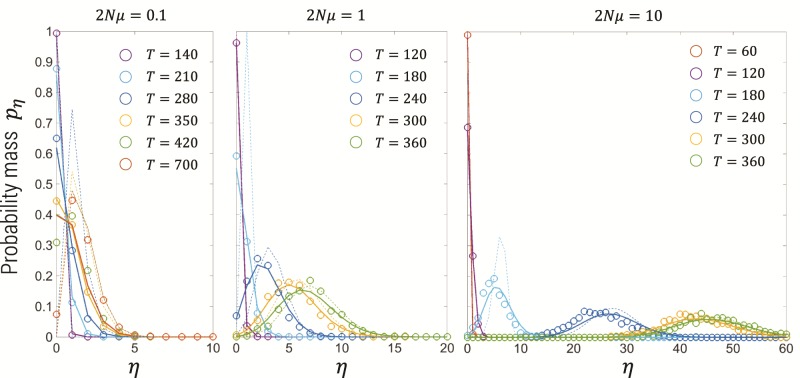
Distribution of the number of origins for simulations with various mutation rates for $N=10^{8}$ and *s *=* *0.05 (open circles) compared with theory in this article equation (12) and (7) (solid lines) and Ewens’ sampling formula (dotted lines), both with *n_s_* = 1,000. For the mutation rates 2Nμ={0.1,1,10}, the corresponding typical fixation time (eq. 5) is $t^{*}\approx \{370,320,280\}$ generations.

We also examine the distribution of the number of origins in figure 4 from Wright–Fisher simulations (1,000 replicates) at a population size $N=10^{8}$, selection coefficient *s *=* *0.05, and mutation rates 2Nμ={0.1,1,10}. The theory presented in this article describes the distribution very well for all times up to and including fixation. On the other hand, Ewens’ sampling framework predicts in a sample of *n_s_* neutral alleles that the distribution of the number of distinct mutant alleles *η* is

(14) $$p(\eta |N,\mu ,n_{s})=\frac{\theta^{\eta }\left[\begin{array}{c} n_{s}\\ \eta \end{array}\right]}{\theta^{\left(n_{s}\right)}},$$

where $\left[\begin{array}{c} n\\ k \end{array}\right]$ is the unsigned Stirling number of the first kind, which is a combinatorial factor which arises in the expansion of the rising factorial $\theta^{\left(n\right)}=\sum_{k=0}^{n}\left[\begin{array}{c} n\\ k \end{array}\right]\theta^{k}=\theta (\theta +1)(\theta +2)\ldots (\theta +n-1)$. However, if the mutant allele has not fixed then the probability distribution of *η* mutants alleles is the convolution of equation (14) with a binomial distribution that in a sample of size *n_s_* we see *n_x_* mutant alleles given a frequency *x*(*t*) of the mutant population. This convolution has no known closed-form solution and for large sample sizes is computationally intensive. In figure 4, the dotted lines are a plot of Ewens’ theory equation (14) without this convolution and *n_s_* replaced in equation (14) by $n_{s}x(t)$ (calculated in Mathematica [Wolfram Research, Inc. 2018]) and as expected it does poorly when the mutant has not yet fixed and is quite accurate at later times when the mutant is near or at fixation. When the mutant allele is at fixation, the semideterministic likelihood of this article and that from Ewens’ formula are closely matched (fig. 7).

## Parameter Estimation

### Haploid

**Fig. 5.**
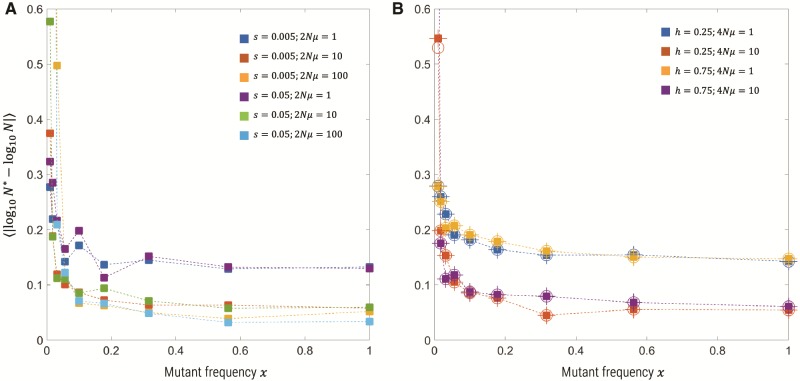
${\;\text{log}\;}_{10}$-error in estimating the true effective population size, for (*A*) haploid populations with $N=10^{8}$, (*B*) diploid populations with $N=5\times 10^{7}$, for various selection coefficients, mutation rates, and dominance coefficients (diploid only) from Wright–Fisher simulations (100 replicates for each parameter combination). (*A*) We use equations (12) and (7) to determine the maximum likelihood estimate. (*B*) For the diploid population, we use the same Poisson likelihood function, but with mean given by equations (13) and (14) in the Supplementary Information, where we assume perfect knowledge of *T* (squares) and also compare to the case where we have a systematic error in our knowledge of *T*, where the true time is T/2 instead *T* (circles), and we see the estimates are unchanged. In addition, for the diploid population we use the haploid likelihood function (eqs. 13 and 7) with θ=4Nμ to estimate *N* (plus signs) and find again excellent agreement.

As described above, the semideterministic theory calculates the likelihood function for the number of observed independent origins, and we find it is only a function of 2Nμ, the frequency of the mutant population at the time of sampling *x*(*T*) and the sample size $n_{s}=1/x_{s}$. Typically, the mutation rate will have been independently determined, and so we can determine a maximum likelihood estimate of *N* given knowledge of the *n_s_* and *x*(*T*), which can be estimated from the sample. In figure 5*A* is the ${\;\text{log}\;}_{10}$-error of this estimation process using 100 replicate Wright–Fisher simulations, with sample size *n_s_* = 1,000, where the true *N* is known. We see that for mutant frequencies *x *>* *0.1, the error of our estimate $N^{*}$ is always less than a factor of $10^{0.2}\approx 1.6$, which means the effective population size is accurately determined to much less than an order of magnitude. Moreover, the accuracy increases for increasing 2Nμ, where it is <$10^{0.1}\approx 1.3$ for 2Nμ≥10.

### Diploid

We can also accurately estimate the effective population size from diploid simulations. As described in the Supplementary Material online, we extend the semideterministic theory to the diploid case with incomplete dominance (0<h<1) by using the exact implicit solution *t*(*x*) for how the frequency *x* of the mutant allele changes over time to calculate time of establishment of the last mutant to be sampled at some later time *T*. This is then used to calculate the likelihood function p(η|N,s,h,μ), where we assume a known mutation rate. We are still left with having to jointly estimate *N*, *s*, and *h* in the diploid case. However, we expect that the dependence on *h* and *s* will be weak (Pennings and Hermisson 2006a), although it is not straightforward to show this explicitly, as in the haploid case, where there is no dependence on *s*, even before fixation. To show this, we use the implicit relation (eq. 2, Supplementary Material online) to numerically estimate $s^{*}$ that gives *t*(*x*) = *T*, where we assume perfect knowledge of the dominance coefficient *h*. We see in figure 5*B* that the estimate of the effective population size from diploid simulations has a similar accuracy as the haploid simulations and is robust to knowledge of the exact time selection sets in *T*; the error is taken up in the estimate of *s* (not shown). We also use the haploid semideterministic theory to estimate the effective population size, using θ=4Nμ in equation (13) to account for double the number of chromosomes, shown by plus signs in figure 5*B*; again we see that the estimate of *N* is identical using the haploid method for a given set of parameters, *s*, *h*, and *μ*. Both the robustness of estimates to the exact knowledge of *T* and that the haploid theory gives identical estimates indicates that the direct dependence on *s* and *h* is very weak or nonexistent, at least for weak absolute selection (Pennings and Hermisson 2006a).

### Haploid with Preexisting Mutations

**Fig. 6.**
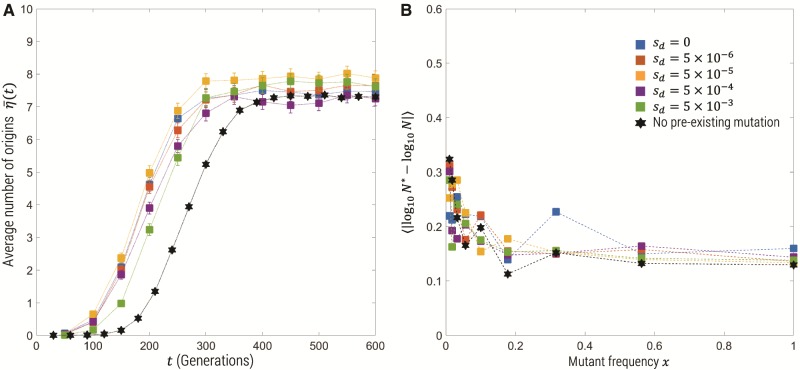
Mean number of origins for haploid simulations with preexisting mutations (*A*), where the black hexagram symbols represent simulations without preexisting simulations, and (*B*) ${\;\text{log}\;}_{10}$-error in maximum likelihood estimate of the true effective population size $N=10^{8}$ from Wright–Fisher simulations with various values of the deleterious selection coefficient *s_d_* (100 replicates for each parameter combination).

Finally, we examine the effect that preexisting mutations have on our estimate of the effective population size. We run simulations such that for times $T_{d}<t<0$ the mutant allele has a negative selection coefficient $s=-s_{d}$, where $2Ns_{d}=\{0,10^{3},10^{4},10^{5},10^{6}\},\;T_{d}=-1,000$ generations and $N=10^{8}$, *s *=* *0.05 and 2Nμ=1. The mean number of origins $\bar{\eta }$ is plotted in figure 6*A*, for the various values of *s_d_* as well as for the case of no preexisting mutations (black hexagram symbols); we see that as the mutant allele becomes increasingly neutral before positive selection sets in, the number of origins is larger, except for long times where the plateau of $\bar{\eta }$ is approximately independent of *s_d_*. This suggests the overall effect of preexisting mutations is to cause a time advance on the number of origins. This again would suggest that the estimate of effective population size should be robust to preexisting mutations, which we see to be the case in figure 6*B*, where the error in estimating *N* using equation (13) for the mean of the Poisson likelihood function is roughly independent of *s_d_* and very similar to assuming no preexisting mutations (black hexagrams).

## Application to Data from Ag1000 Project

**Fig. 7.**
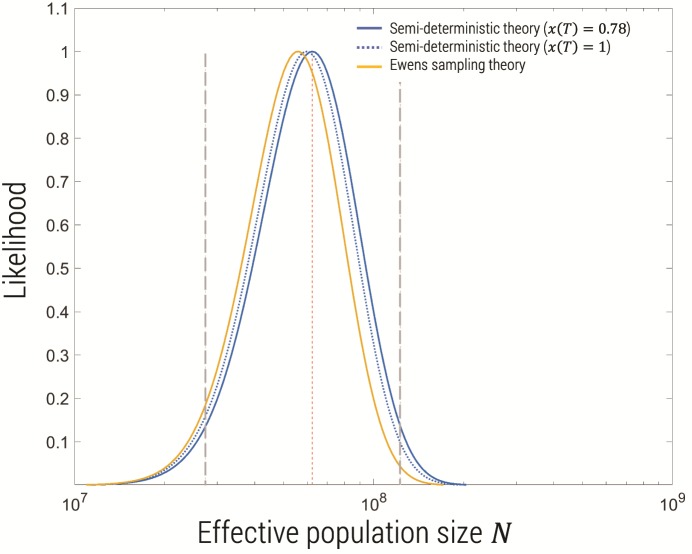
Likelihood (normalized) of the number of origins as function of effective population size given an observed number *η*  =  10 and samples size *n_s_* = 1,530 chromosomes, corresponding to that found for the Ag1000 project (*Anopheles gambiae* 1000 Genomes Consortium 2017) for the *Vgsc* resistance locus. As shown in the legend, the semideterministic theory in this article, assuming a current day frequency of *x *=* *0.78 (as observed) is compared with assuming *x *=* *1 and the Ewens’ sampling theory equation (14), which only has applicability for *x *=* *1. The 95% confidence intervals (gray dotted lines) and maximum likelihood effective population size (red dotted line) are shown for the semideterministic likelihood function with *x *=* *0.78.

Recently published data from the Ag1000 project have extensive population level sampling of the genomes of mosquitoes across sub-Saharan Africa (*Anopheles gambiae* 1000 Genomes Consortium 2017). The gene for the voltage-gated sodium channel (*Vgsc*) is known to have at least two single nucleotide mutations in the same codon that confer resistance to insecticides, L995S (2984T>C) and L995F (2985A>T), and phylogenetic analysis of this gene reveal ten haplotype clusters (fig. 4 in *Anopheles gambiae* 1000 Genomes Consortium [2017]) with a current mutant frequency of x≈0.78 determined directly from the data. If we assume either mutation is required for resistance, this gives a mutation rate of $\mu \approx 6\times 10^{-9}$, assuming a base-pair mutation rate of $3\times 10^{-9}$, which is based on a recent accurate estimate from *Drosophila* (Keightley et al. 2014), as the mutation rate has not been directly measured for *A. gambiae*. Applying the haploid algorithm to this data, using θ=4Nμ in equation (13) (accounting for the factor of 2 between chromosomes and individuals), and *n_m_* = 1,193 (given a sample size of *n_s_* = 1,530 chromosomes from 765 mosquitoes), gives an estimate of θ=1.5(0.66,3), which corresponds to an effective population size $N=6.2\times 10^{7}\;(2.7\times 10^{7},1.2\times 10^{8})$, where the values in brackets are the 95% confidence intervals (2 ln units from max likelihood), as shown in the plot of the likelihood function in figure 7. This estimate is almost 2 orders of magnitude greater than that of $N\approx 10^{6}$ from a nucleotide diversity π∼0.01. In the same article, the authors use the more sophisticated “stairway” plot (Liu and Fu 2015) and ∂a∂i (Gutenkunst et al. 2009) method to estimate population history and find most recent effective population sizes of order $N\approx 10^{7}$, which is roughly six times less than our estimate.

Note that we can also apply the method to each resistance mutant separately L995S and L995F, which have frequencies of ≈0.28 and ≈0.5, and five independent origins each, which assuming a single base-pair mutation rate of $\approx 3\times 10^{-9}$ for each of these, gives the following estimates of effective population size $N=6.6\times 10^{7}\;(1.9\times 10^{7},1.7\times 10^{8})$, and $N=6.0\times 10^{7}\;(1.8\times 10^{7},1.5\times 10^{8})$, respectively, where the values in brackets, are again the 95% confidence intervals. We see the estimates based on each single nucleotide polymorphism are consistent with the estimate above based on both single nucleotide polymorphisms, but, as expected, with larger confidence intervals.

However, it is known that in many sub-Saharan regions mosquitoes undergo seasonal demographic changes, where the population size changes between wet and dry seasons by up to a peak-to-trough factor of $\varphi =N_{\text{max}}/N_{\text{min}}=100$ (Minakawa et al. 2002; Mabaso et al. 2007; Bomblies et al. 2009; Walker et al. 2013), where *N*_max_ and *N*_min_ are the maximum and minimum of the population size. To check the impact of demographic changes on our population size estimates, we ran simulations for a mutant with *s *=* *0.05, with an oscillating population size $N(t)=\frac{1}{2}(N_{\text{max}}+N_{\text{min}})+\frac{1}{2}(N_{\text{max}}-N_{\text{min}})\;\text{sin}(2\pi t/\Delta T)$, with a period of ΔT=12 generations, which is ∼1 year and much shorter than the expected time to fixation of the mutant of ∼300 generations (eq. 5). The simulations were performed with various peak-to-trough ratios $\varphi =N_{\text{max}}/N_{\text{min}}=\{10,100,1000\}$ and with two constraints: 1) that the geometric mean ${\langle N\rangle }_{G}=\sqrt{N_{\text{max}}N_{\text{min}}}=N=10^{8}$ and 2) that the harmonic mean ${\langle N\rangle }_{H}=2{\left(N_{\text{max}}^{-1}+N_{\text{min}}^{-1}\right)}^{-1}=N=10^{8}$. Simulations with constrained arithmetic mean were also performed but are not shown.

**Fig. 8.**
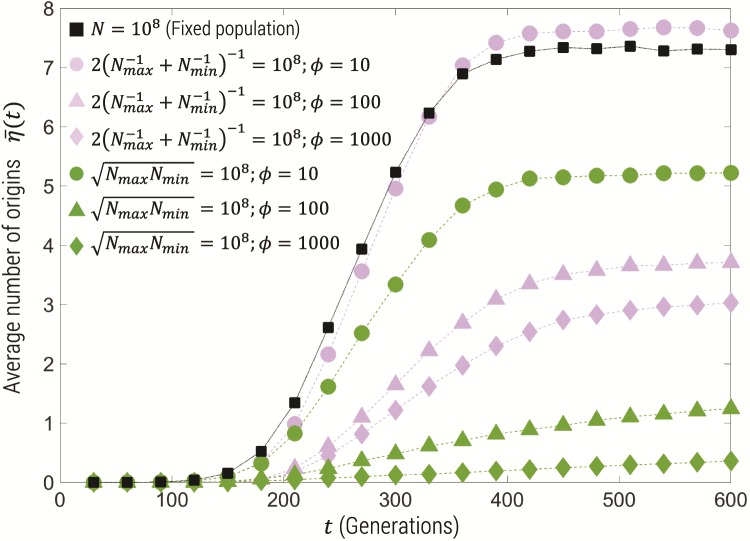
The mean number of origins from Wright–Fisher simulations (1,000 replicates) for oscillating population size with period ΔT=12 generations, selection coefficient *s *=* *0.05, 2Nμ=1, and with the geometric mean (green) and harmonic mean (purple) of *N*_max_ and *N*_min_ constrained to $\sqrt{N_{\text{max}}N_{\text{min}}}=2{\left(1/N_{\text{max}}+1/N_{\text{min}}\right)}^{-1}=N=10^{8}$, for different peak-to-trough ratios. Black squares represent constant population size simulations.

Overall, we see in figure 8 that constraining the harmonic mean of the maximum and minimum population size for a given φ gives fewer origins than simulations with a constant population size, and more origins than simulations that constrain the geometric mean, the exception being for φ=10 where the number is slightly larger, but roughly equal, to the constant population size case. This means we can broadly say that using the constant population size theory to estimate $N^{*}$ will give a relatively tight *lower bound* on the true harmonic mean (with weaker lower bounds on the geometric mean, and arithmetic mean as discussed below). Given the simple relation between the harmonic mean and maximum and minimum population sizes we can derive expressions for a lower bound on *N*_min_ and *N*_max_ given an estimate of $N^{*}$ and φ:

(15) $$\begin{array}{c} N_{\text{max}}>\frac{N^{*}}{2}(1+\varphi )\\ N_{\text{min}}>\frac{N^{*}}{2}(1+1/\varphi ) \end{array}.$$

This is true for any value of φ. On the other hand, from figure 8, we can see for φ=100 that the number of origins due to the harmonic constraint is approximately one half the origins assuming a constant effective population size, so as equation (13) is almost linear in *θ*, with only a weak logarithmic nonlinearity, the true harmonic mean can be estimated as ${\langle N\rangle }_{H}\approx 2N^{*}$ (simulations with harmonic mean constrained to $2N^{*}$ confirm this—not shown). The field data (Minakawa et al. 2002; Mabaso et al. 2007; Bomblies et al. 2009; Walker et al. 2013) suggest φ≤100, which means ${\langle N\rangle }_{H}<2N^{*}$. We can then upper bound the maximum and minimum effective population sizes as $N_{\text{max}}<N^{*}(1+\varphi )$ and $N_{\text{min}}<N^{*}(1+1/\varphi )$, which is only true specifically for φ≤100.

Altogether, this gives the following bounds on *N*_max_ and *N*_min_: $3.1\times 10^{9}\leq N_{\text{max}}\leq 6.2\times 10^{9}$ and $3.1\times 10^{7}\leq N_{\text{min}}\leq 6.2\times 10^{7}$, using the estimate above of $N^{*}=6.2\times 10^{7}$. Using these bounds, we can then put a bound on the arithmetic mean $\langle N\rangle =\frac{1}{2}(N_{\text{max}}+N_{\text{min}})$, as $1.6\times 10^{9}\leq \langle N\rangle \leq 3.1\times 10^{9}$. Note that this result needs a little care in interpretation, since as seen in figure 8 for φ=10 the harmonic constraint gives slightly greater independent origins, however, it is with near equality and within the errors of these estimates. Simulations that constrain the arithmetic mean of the maximum and minimum population sizes show that the number of origins monotonically decreases with increasing φ, but are significantly less than even the constrained geometric mean case (not shown). This means our estimate $N^{*}$ will be less than the arithmetic mean for all φ, but as with the geometric mean, the equivalent to equation (15) would provide a much weaker *lower* bound on *N*_max_ and *N*_min_.

## Discussion

Estimating the recent effective population size is of paramount importance to understanding and predicting the evolutionary dynamics of natural populations. As has been previously suggested (Karasov et al. 2010), methods that estimate effective population size based on nucleotide diversity are likely to give estimates which are much smaller than the current day census size, as such metrics are dominated by historical population bottlenecks. Although methods based on linkage disequilibrium can detect recent effective population sizes, they tend to be limited to small populations (Waples and Do 2010). In addition, methods that estimate demographic histories tend to be computationally complicated and with limited range of applicability, such as only detecting long-term variations (Pybus et al. 2000; Gutenkunst et al. 2009; Liu and Fu 2015) or limited to small population sizes (Browning and Browning 2015). However, a genomic region undergoing current selection should leave a signature which represents an effective population size more representative of the census size during the sweep (Karasov et al. 2010). When the mutational input into a population is large 2Nμ>1, we expect a signature of a selective sweep will be a large diversity of haplotype backgrounds, due to multiple and recurrent independent instances of the same mutation that is under positive selection; such a sweep has been termed a soft sweep as multiple rather than a single haplotype dominate the sweep (Hermisson and Pennings 2005). Although Pennings and Hermisson seminal work (Pennings and Hermisson 2006a, 2006b) laid out much of our understanding of soft sweeps within a coalescence framework, many quantities like the likelihood of the number of origins, particularly when the mutant population has not yet fixed, are not straightforward to calculate numerically.

In this article, we have presented a simple semideterministic haploid forward-time theory of the number of independent origins of a recurrent mutation. We show that the distribution of the number of origins is very closely approximated by a Poisson distribution with a mean number of origins that has an exact and simple closed-form solution for the haploid case, which is independent of the selection coefficient and the age of the allele, and only depends on 2Nμ, the sample size and the current day mutant frequency. We show it works robustly for diploid populations with incomplete dominance, and whether or not mutations are preexisting in the population before the selection pressure arose.

Our forward-time semideterministic theory also provides an intuitive insight into the dynamics of soft sweeps, where it is clear there is a demarcation between the stochastic and deterministic stages for each haplotype contributing to a soft sweep. New origins are generated by recurrent mutation, and these must establish by growing to a frequency where deterministic selection outweighs drift; thereafter growth is approximately deterministic of each independent mutant. The deterministic part of the theory shows that at sufficiently large population sizes the growth of each recurrent mutant is just a scaling of the overall mutant population and grows logistically, where other mutants “crowd-out” the growth of a particular mutant; once the wild type is extinct new mutants cannot arise, and growth of each recurrent mutant is zero, so this structure is effectively frozen, which is confirmed by simulation up to small fluctuations due to drift. Including drift in this picture means that this frozen structure is only temporary as drift will take of order *N* generations to act. This is seen in the simulations at even a moderate population size of $N=10^{6}$, where drift can act on the small frequency variants causing a decrease in independent origins for long times; however, for very large populations $N\gg 10^{7}$ there is a stable plateau as predicted by the theory. This suggests that Ewens’ sampling theory and the calculation in this article will not be valid for small populations after fixation of the mutant, since the supply of mutants has been switched off; therefore the semideterministic approach in this article will be limited to times at or before fixation for small population sizes.

The framework of this semideterministic theory also makes clear why selection should have little effect on the plateau number of origins, as the rate of establishment is proportional to the *s*, whereas the time window over which new origins can be generated is proportional to the lifetime of the wild type, which scales as 1/s, giving a number of origins that is independent of *s*. In addition, our result for the mean number of origins shows further that it is only dependent on the selection coefficient through the frequency of the mutant population, and in particular on the ratio of the number of mutants in the sample to the number of new mutants that enter every generation (2Nμ). Surprisingly, as found by Pennings and Hermisson (2006a), the number of origins does not depend on the exact sample path (history of the population frequency) of the mutant; here we see further that the number of origins only depends on the frequency of the mutant at a given time.

Finally, we estimated the effective population size of *A. gambiae* and *Anopheles coluzzii* to be $N\approx 6.2\times 10^{7}$ using data from the 1000Ag project (*Anopheles gambiae* 1000 Genomes Consortium 2017), which is roughly 2 orders of magnitude larger than estimated using the same underlying data from nucleotide diversity and much closer to what is likely to be the census population size in recent history. This supports simple calculations of Karasov et al. (2010), which suggested values of effective population size derived from nucleotide diversity are too small to explain adaptation of resistance alleles or the occurrence of multiple resistance haplotypes for the *Ace* gene in *Drosophila melanogaster*. Here, we have provided a very simple and robust method to quantify this effect.

The demographic history of *Anopheles* has also been estimated from the 1000Ag project data (*Anopheles gambiae* 1000 Genomes Consortium 2017) using the “stairway” plot (Liu and Fu 2015) and ∂a∂i (Gutenkunst et al. 2009) methods, giving a recent population size of roughly $N\approx 10^{7}$, greater than the nucleotide diversity estimate, but smaller than our estimate. A possible reason for this discrepancy is that these methods tend to detect long-term demographic changes, so that the difference could represent recent population growth in the past 100 years. However, there are reasons to be uncertain about these estimates; the estimates in *Anopheles gambiae* 1000 Genomes Consortium (2017) are based on applying each of these methods to data from each geographic region, whereas the estimate here is based on data from all geographic regions in the Ag1000 data. In the completely panmictic case, the estimate in each region should agree with the estimate based on pooling the data, but as discussed below if there is spatial structure then the relation between the two estimates would not be straightforward. There is also good reason to suggest there may have been a reduction in effective population size due to action of insecticides (Athrey et al. 2012; O’Loughlin et al. 2016).

More generally, a recent population expansion would further lead to our method underestimating the current day population size. In an expanding population, there would be a maximum in the number of wild-type individuals that produce independent origins at around the time $t^{*}$, since at very early times the overall population size is small and at longer times than $t^{*}$ the wild type is near extinction. Therefore, if selection is particularly strong and $t^{*}$ occurs far in the past compared with the current age of the allele, this would be a very large underestimate, as the number of observed origins would be controlled by a time when the census was very small. For the *Vgsc* gene of *A. gambiae* given that the current day mutant frequency x(T)=0.78, and T≈960 generations (assuming that insecticides were introduced about 80 years ago), we can use equation (3) to numerically find the best fit selection coefficient as $s^{*}\approx 0.017$ with the constraint that $\mu =6\times 10^{-9}$. Then, using equation (5), we calculate $t^{*}\approx 885$ generations; this is the recent past, which suggests our estimate $N^{*}$ should not be too great an underestimate. On the other hand, if there has been a recent decline in population numbers then this would have an opposite effect, where our method would overestimate the effective population size due the overall number of origins being dominated for times $t<t^{*}$, when the population was larger in the past. Again, with our estimate of $t^{*}$ being in the recent past suggests the error will be small. Additionally, as discussed in Pennings et al. (2014), if there are preexisting mutations then the population size estimate would be influenced by the size before insecticides were introduced.

It is also known that the *Anopheles* populations undergo seasonal demographic fluctuations with peak-to-trough population sizes of order 10–100 (Minakawa et al. 2002; Mabaso et al. 2007; Bomblies et al. 2009; Walker et al. 2013). To investigate the effect of such fluctuations on our population size estimates, we performed simulations of oscillating population sizes over time for peak-to-trough factors <1,000. These results showed that a constant population size estimate will tend to underestimate the harmonic mean of the maximum and minimum of the population size for large peak-to-trough ratios φ. In addition, the simulations show for φ=100, the average number of origins is approximately one half. Together, this allowed quantification of bounds on the maximum and minimum population size giving $3.1\times 10^{9}\leq N_{\text{max}}\leq 6.2\times 10^{9}$ and $3.1\times 10^{7}\leq N_{\text{min}}\leq 6.2\times 10^{7}$, assuming a peak-to-trough ratio of φ≤100, as suggested by the field data (Minakawa et al. 2002; Mabaso et al. 2007; Bomblies et al. 2009; Walker et al. 2013). This then suggests a mean (arithmetic) population size bounded as $1.6\times 10^{9}\leq \langle N\rangle \leq 3.1\times 10^{9}$.

One might ask if seasonal oscillating demographics could alone explain the discrepancy between the *N* estimated from nucleotide diversity and our larger estimate here. Our results in figure 8 suggest that for increasing peak-to-trough ratio, we would expect to underestimate the harmonic, geometric and arithmetic mean (arithmetic mean not shown), and so given our constant population size estimate using equation (13) of $N^{*}=6.2\times 10^{7}$, we in fact would expect the discrepancy with respect to the nucleotide diversity estimate to be even larger and this cannot in itself explain the discrepancy. However, this is comparing to the estimate of *N* from *π* assuming a constant/fixed population size. For an oscillating population, the nucleotide diversity will be controlled by the harmonic average of the effective population size over a cycle (Wright 1938; Charlesworth 2009), which can be shown to be given by the geometric mean of the maximum and minimum of the sinusoidal demographic variation (i.e., ${\left[\frac{1}{\Delta T}\int_{0}^{\Delta T}N{\left(t\right)}^{-1}dt\right]}^{-1}=\sqrt{N_{\text{max}}N_{\text{min}}}={\langle N\rangle }_{G}$). This means for different values of peak-to-trough factors φ with the same geometric mean $\sqrt{N_{\text{max}}N_{\text{min}}}$, the nucleotide diversity should be unchanged, on the other hand the simulations in figure 8 show that we should observe fewer origins for increasing φ; this is inconsistent with observations, as fewer origins corresponds to an *underestimate* of the geometric mean, which is the effective population size estimated by nucleotide diversity. In other words, oscillating demographics with an unchanging mean would lead to the nucleotide diversity estimate of *N* to be *greater* than the value estimated from number of origins assuming a nonoscillating and fixed population size. We observe the opposite, which suggests there is another mechanism by which nucleotide diversity has been suppressed, such as historical and sustained bottlenecks.

The results of these oscillating demographic simulations are in contrast to those of Wilson et al. (2014), which showed that the probability of a soft sweep in a sample size of 2 only depends on the cycle-averaged harmonic mean, when demographic oscillations are fast. As mentioned above the cycle-averaged harmonic mean is just the geometric mean of the maximum and minimum population sizes; however, our results show different peak-to-trough ratios give significantly different numbers of independent origins for the same geometric mean. This suggests the probability of a soft sweep in a sample size of 2 is a weak measure of the diversity of haplotypes compared with the number of independent origins.

Our estimation also makes the assumption that the populations are well mixed or panmictic and constant over time, which clearly requires testing regarding the Ag1000 data, which consists of the sequences of individuals collected over the wide spatial region of sub-Saharan Africa. As discussed by Ralph and Coop (2010), we would expect our results to be accurate in the limit of strong long-range or nonlocal dispersal, which mimics the panmictic approximation; on the other hand, if local migration is strong, spatial structure of the populations would tend to give a larger number of origins compared with the panmictic case, which would suggest our method would overestimate the effective population size needed to explain an observed number of origins. In other words, it is possible that spatial structure could account partially or wholly for the large number of origins observed in natural populations of *A. gambiae* and *Anopheles coluzzii*. Further theory and simulations will be needed to test this hypothesis.

## Supplementary Material

Supplementary data are available at *Molecular Biology and Evolution* online.

## Supplementary Material

msz081_Supplementary_Data

## References

[msz081-B1] Anderson TJ, Nair S, McDew-White M, Cheeseman IH, Nkhoma S, Bilgic F, McGready R, Ashley E, Phyo AP, White NJ, et al 2017 Population parameters underlying an ongoing soft sweep in Southeast Asian malaria parasites. Mol Biol Evol. 34 1:131–144. 28025270 10.1093/molbev/msw228 PMC5216669

[msz081-B2] Anopheles gambiae 1000 Genomes Consortium. 2017 Genetic diversity of the African malaria vector *Anopheles gambiae*. Nature 552 7683:96. 29186111 10.1038/nature24995 PMC6026373

[msz081-B3] Athrey G, Hodges TK, Reddy MR, Overgaard HJ, Matias A, Ridl FC, Kleinschmidt I, Caccone A, Slotman MA. 2012 The effective population size of malaria mosquitoes: large impact of vector control. PLoS Genet. 8 12:e1003097. 23271973 10.1371/journal.pgen.1003097 PMC3521722

[msz081-B4] Bollback JP, York TL, Nielsen R. 2008 Estimation of 2nes from temporal allele frequency data. Genetics 179 1:497–502. 18493066 10.1534/genetics.107.085019 PMC2390626

[msz081-B5] Bomblies A, Duchemin J-B, Eltahir EA. 2009 A mechanistic approach for accurate simulation of village scale malaria transmission. Malaria J. 8 1:223. 10.1186/1475-2875-8-223 PMC2761400 19799793

[msz081-B6] Browning S, Browning B. 2015 Accurate non-parametric estimation of recent effective population size from segments of identity by descent. Am J Hum Genet. 97 3:404–418. 26299365 10.1016/j.ajhg.2015.07.012 PMC4564943

[msz081-B7] Charlesworth B. 2009 Effective population size and patterns of molecular evolution and variation. Nat Rev Genet. 10 3:195–205. 19204717 10.1038/nrg2526

[msz081-B8] Desai MM, Fisher DS. 2007 Beneficial mutation selection balance and the effect of linkage on positive selection. Genetics 176 3:1759–1798. 17483432 10.1534/genetics.106.067678 PMC1931526

[msz081-B9] Ewens WJ. 2010 Mathematical population genetics: 1. A theoretical introduction. New York: Springer.

[msz081-B10] Feder AF, Kline C, Polacino P, Cottrell M, Kashuba ADM, Keele BF, Hu S-L, Petrov DA, Pennings PS, Ambrose Z. 2017 A spatio-temporal assessment of simian/human immunodeficiency virus (shiv) evolution reveals a highly dynamic process within the host. PLoS Pathog. 13 5: e1006358. 10.1371/journal.ppat.1006358 PMC5444849 28542550

[msz081-B11] Fisher RA. 1930 The genetical theory of natural selection. Oxford: Oxford University Press.

[msz081-B12] Gutenkunst RN, Hernandez R, Williamson S, Bustamante C. 2009 Inferring the joint demographic history of multiple populations from multidimensional SNP frequency data. PLoS Genet. 5 10:e1000695. 10.1371/journal.pgen.1000695 PMC2760211 19851460

[msz081-B13] Hermisson J, Pennings PS. 2005 Soft sweeps: molecular population genetics of adaptation from standing genetic variation. Genetics 169 4:2335–2352. 15716498 10.1534/genetics.104.036947 PMC1449620

[msz081-B14] Karasov T, Messer PW, Petrov DA. 2010 Evidence that adaptation in drosophila is not limited by mutation at single sites. PLoS Genet. 6 6:e1000924. 20585551 10.1371/journal.pgen.1000924 PMC2887467

[msz081-B15] Keightley PD, Ness RW, Halligan DL, Haddrill PR. 2014 Estimation of the spontaneous mutation rate per nucleotide site in a *Drosophila melanogaster* full-sib family. Genetics 196 1:313–320. 24214343 10.1534/genetics.113.158758 PMC3872194

[msz081-B16] Khatri BS. 2016 Quantifying evolutionary dynamics from variant-frequency time series. Sci Rep. 6: 32497. 10.1038/srep32497 PMC5018853 27616332

[msz081-B17] Kimura M. 1962 On the probability of fixation of mutant genes in a population. Genetics 47:713–719. 14456043 10.1093/genetics/47.6.713 PMC1210364

[msz081-B18] Liu X, Fu Y-X. 2015 Exploring population size changes using SNP frequency spectra. Nat Genet. 47 5:555–559. 25848749 10.1038/ng.3254 PMC4414822

[msz081-B19] Mabaso MLH, Smith T, Ross A, Craig M. 2007 Environmental predictors of the seasonality of malaria transmission in Africa: the challenge. Am J Trop Med Hyg. 76 1:33–38. 17255225

[msz081-B20] Messer PW, Neher RA. 2012 Estimating the strength of selective sweeps from deep population diversity data. Genetics 191 2:593–605. 22491190 10.1534/genetics.112.138461 PMC3374320

[msz081-B21] Minakawa N, Sonye G, Mogi M, Githeko A, Yan G. 2002 The effects of climatic factors on the distribution and abundance of malaria vectors in Kenya. J Med Entomol. 51 4:833–841. 10.1603/0022-2585-39.6.833 12495180

[msz081-B22] O’Loughlin SM, Magesa SM, Mbogo C, Mosha F, Midega J, Burt A. 2016 Genomic signatures of population decline in the malaria mosquito *Anopheles gambiae*. Malaria J. 15 1:182. 10.1186/s12936-016-1214-9 PMC4806450 27013475

[msz081-B23] Pennings PS, Hermisson J. 2006a Soft sweeps II—molecular population genetics of adaptation from recurrent mutation or migration. Mol Biol Evol. 23 5:1076–1084. 16520336 10.1093/molbev/msj117

[msz081-B24] Pennings PS, Hermisson J. 2006b Soft sweeps III: the signature of positive selection from recurrent mutation. PLoS Genet. 2 12:e186. 17173482 10.1371/journal.pgen.0020186 PMC1698945

[msz081-B25] Pennings PS, Kryazhimskiy S, Wakeley J. 2014 Loss and recovery of genetic diversity in adapting populations of HIV. PLoS Genet. 10 1:e1004000. 24465214 10.1371/journal.pgen.1004000 PMC3900388

[msz081-B26] Petkova D, Novembre J, Stephens M. 2016 Visualizing spatial population structure with estimated effective migration surfaces. Nat Genet. 48 1:94–100. 26642242 10.1038/ng.3464 PMC4696895

[msz081-B27] Pybus OG, Rambaut A, Harvey PH. 2000 An integrated framework for the inference of viral population history from reconstructed genealogies. Genetics 155 3:1429–1437. 10880500 10.1093/genetics/155.3.1429 PMC1461136

[msz081-B28] Ralph PL, Coop G. 2010 Parallel adaptation: one or many waves of advance of an advantageous allele? Genetics 186 2:647–668. 20660645 10.1534/genetics.110.119594 PMC2954473

[msz081-B29] Walker M, Winskill P, Basáñez MG, Mwangangi JM, Mbogo C, Beier JC, Midega JT. 2013 Temporal and micro-spatial heterogeneity in the distribution of *Anopheles* vectors of malaria along the Kenyan coast. Parasit Vectors 6 1:311. 24330615 10.1186/1756-3305-6-311 PMC3843567

[msz081-B30] Waples RS, Do C. 2010 Linkage disequilibrium estimates of contemporary ne using highly variable genetic markers: a largely untapped resource for applied conservation and evolution. Evol Appl. 3 3:244–262. 25567922 10.1111/j.1752-4571.2009.00104.x PMC3352464

[msz081-B31] Wilson BA, Petrov DA, Messer PW. 2014 Soft selective sweeps in complex demographic scenarios. Genetics 198 2:669–684. 25060100 10.1534/genetics.114.165571 PMC4266194

[msz081-B32] Wolfram Research, Inc. 2018 Mathematica, version 11.3. Champaign (IL).

[msz081-B33] Wright S. 1931 Evolution in Mendelian populations. Genetics 16 2:97–159. 17246615 10.1093/genetics/16.2.97 PMC1201091

[msz081-B34] Wright S. 1938 Size of population and breeding structure in relation to evolution. Science 87:430–431.

[msz081-B35] Zanini F, Brodin J, Thebo L, Lanz C, Bratt G, Albert J, Neher RA. 2015 Population genomics of intrapatient HIV-1 evolution. eLife 4:e11282. 26652000 10.7554/eLife.11282 PMC4718817

