# Supplementary material for: Robust Estimation of Recent Effective Population Size from Number of Independent Origins in Soft Sweeps
Source: Mol Biol Evol. 2019 Apr 9;36(9):2040–52. doi: 10.1093/molbev/msz081 (PMC6736332; doi:10.1093/molbev/msz081)
Supplement: msz081_Supplementary_Data [file msz081_supplementary_data.zip › MBE-Final_SI_3rdApril2019.pdf]

Supplementary Information
Robust estimation of recent effective population size from number of independent origins in soft sweeps

Bhavin S. Khatri
Department of Life Sciences, Imperial College London, Silwood Park, Ascot, SL5 7PY, U.K. and
The Francis Crick Institute, 1 Midland Road, London, NW1 1AT, U.K.[*]

Austin Burt
Department of Life Sciences, Imperial College London, Silwood Park, Ascot, SL5 7PY, U.K.

MEAN NUMBER OF ORIGINS FOR DIPLOID POPULATION

In the diploid case an exact analytical form for the total mutant frequency $x(t)$ does not exist, however, we can make progress by using the implicit solution $t(x)$, which does have an exact analytical form. The differential equation determining the change in frequency of the mutant population, including diploidy is:

$$\frac{\mathrm{d}x}{\mathrm{d}t} = s(h + (1-2h)x)x(1-x) + \mu(1-x). \qquad \text{(S1)}$$

Using partial fractions the implicit solution is:

$$t(x) = \frac{1}{sh(1+\alpha+\beta)}\left(\frac{1+2\alpha}{\sqrt{4\alpha\beta-1}}\tan^{-1}\left(\frac{1+2\alpha x}{\sqrt{4\alpha\beta-1}}\right) - \ln(x-1) + \frac{1}{2}\ln(\alpha x^2 + x + \beta)\right), \qquad \text{(S2)}$$

where $\alpha = \frac{1-2h}{h}$ and $\beta = \frac{\mu}{sh}$. Using a similar argument as the haploid case, we can find the frequency of the last or K^{th} mutant (equivalent of Eqn.10 in main text):

$$x_K(T) = \frac{x(T)}{4Nhsx(t_K)}, \qquad \text{(S3)}$$

where here we have used the approximation that the establishment frequency will be dominated by heterozygotes, and be $x_{est} \approx \frac{1}{4Nhs}$. Eqn.S2 is then used to solve for t_K, using as before $x_K(T) = x_s$:

$$t_K = t(x(t_K)) = t\left(\frac{x(T)/x_s}{4Nhs}\right), \qquad \text{(S4)}$$

where $t(x)$ is the implicit function given in Eqn.S2. Similarly, we approximate the probability of establishment as that of the heterozygote, so $p_{est} \approx 2hs$; this gives the inhomogeneous Poisson rate as

$$\alpha(t) \approx 4N\mu hs(1 - x(t)), \qquad \text{(S5)}$$

and so the mean number of origins will be

$$\begin{aligned}\bar{\eta}(T) &= \int_0^{t_K(T)} \alpha(t)\mathrm{d}t \\ &= 4N\mu hs\left(t_K(T) - \int_0^{t_K(T)} x(t)\mathrm{d}t\right).\end{aligned} \qquad \text{(S6)}$$

However, we do not have an explicit form for $x(t)$. Given the implicit form (Eqn.S2) this can be numerically integrated, however, we instead develop an approximation for $x(t)$. This involves approximating the RHS of Eqn.S2, $F(x)$ by a piecewise quadratic, up to a frequency x^*, which is the frequency at which $\frac{\mathrm{d}F}{\mathrm{d}x} = 0$; for $0 \le h \le 1$ this is a reasonable approximation. As this approximate $F(x)$ is quadratic, an exact solution can be found for each region, $0 \le x \le x^*$

* bhavin.khatri@physics.org

and $x^* < x \leq 1$, and is of the form given for the haploid case in Eqn.3 in the main text. These solutions are then matched at the common point of inflexion which occurs at (t^*, x^*). This gives the following solution:

$$x(t) = 1-\kappa+\kappa\Big(x^*(1+\tanh\gamma_1(t-t^*))\Big) \quad \text{for } t < t^* \qquad \text{(S7)}$$

and

$$x(t) = 1-\kappa+\kappa\Big(x^*+(1-x^*)\tanh\gamma_2(t-t^*)\Big) \quad \text{for } t \geq t^* \qquad \text{(S8)}$$

where

$$x^* = \frac{(1-3h)+\sqrt{3h^2+3h(2\mu/s-1)+1-3\mu/s}}{3(1-2h)}, \qquad \text{(S9)}$$

$$\gamma_1 = \frac{sF(x^*)}{x^*} \qquad \text{(S10)}$$

$$\gamma_2 = \frac{sF(x^*)}{1-x^*}, \qquad \text{(S11)}$$

and κ relates to a correction so the solution matches the initial condition $x(0) = x_0$:

$$\kappa = \frac{1-x_0}{1-x^*+x^*\tanh(\gamma_1 t^*)}. \qquad \text{(S12)}$$

These can now be inserted into Eqn.S6 and integrated up to $t_K(T)$, to find the mean number of origins:

$$\bar{\eta}(T) = 4N\mu hs\kappa\Bigg(t_K(1-x^*) - \frac{x^*}{\gamma_1}\ln\left(\frac{\cosh\gamma_1(t_K-t^*)}{\cosh\gamma_1 t^*}\right)\Bigg) \quad \text{for } t_K < t^* \qquad \text{(S13)}$$

$$\bar{\eta}(T) = 4N\mu hs\kappa\Bigg(t_K(1-x^*) + \frac{x^*}{\gamma_1}\ln(\cosh\gamma_1 t^*) - \frac{1-x^*}{\gamma_2}\ln(\cosh\gamma_2(t_K-t^*))\Bigg) \quad \text{for } t_K \geq t^*. \qquad \text{(S14)}$$

The likelihood in the diploid case is then given by the Poisson distribution:

$$p(\eta(T)|N,s,h,\mu) = \frac{\bar{\eta}(T)^\eta}{\eta!}e^{-\bar{\eta}(T)}, \qquad \text{(S15)}$$
